# The Role of Radiotherapy in the Treatment of Small-Cell Lung Cancer

**DOI:** 10.1007/s11864-015-0372-2

**Published:** 2015-10-15

**Authors:** Kaname Nosaki, Takashi Seto

**Affiliations:** Department of Thoracic Oncology, National Kyushu Cancer Center, 3-1-1 Notame, Minami-ku, Fukuoka 811-1395 Japan

**Keywords:** Small-cell lung cancer, Prophylactic cranial irradiation, Thoracic radiotherapy

## Abstract

The standard therapy for limited disease small cell lung cancer (LD-SCLC) is concurrent chemoradiotherapy and prophylactic cranial irradiation (PCI) for those who achieve complete remission (CR) or good partial response (PR) with initial therapy. On the other hand, the standard therapy for extensive disease (ED-SCLC) is chemotherapy only. After the two phase III study conducted by Slotman et al., PCI with/without thoracic radiotherapy (TRT) is also recommended in the treatment of ED-SCLC. However, a Japanese phase III study failed to confirm the benefit of PCI for patients with ED-SCLC. All studies have demonstrated the effectiveness of PCI for preventing brain metastasis, but PCI seems to have a limited influence on OS. In the 2014 edition of the *Guidelines for the Treatment of Lung Cancer* from the Japan Lung Cancer Society (JLCS), use of PCI for patients with ED-SCLC has been changed from “recommended” to “not recommended”. Appropriate selection of patients for PCI with/without TRT is very important. It is hoped that the characteristics of patients for whom PCI with/without TRT should be considered or avoided will be better defined in the future.

## Introduction

Small cell lung cancer (SCLC) has classified as either LD-SCLC or ED-SCLC. LD-SCLC is defined as confined within an ipsilateral hemithorax that can be safely encompasses within “tolerable radiation field” [1]. The standard therapy for LD-SCLC is concurrent chemoradiotherapy and PCI for those who achieve CR or good PR with initial therapy. On the other hand, the standard therapy for ED-SCLC is chemotherapy only. These days, PCI with/without thoracic radiotherapy (TRT) is also recommended in the treatment of ED-SCLC. In this review, we focus on the role of PCI for LD-SCLC, PCI for ED-SCLC, and TRT for ED-SCLC.

## PCI

For patients with SCLC, PCI has been performed since the 1970s for the prevention of brain metastases, which are resistant to systemic chemotherapy alone because of the blood–brain barrier [2]. In 1999, a meta-analysis of clinical studies on the usefulness of PCI was published, revealing that PCI not only reduces the incidence of brain metastasis but also prolongs the survival of patients who achieve CR after initial treatment, irrespective of whether they have LD-SCLC or ED-SCLC [3]. After this report, PCI was performed for patients who achieved CR or good PR with initial therapy and it was also considered for patients with ED-SCLC. In 2007, it was reported that PCI reduces the incidence of brain metastasis and prolongs the survival of patients who respond to their initial treatment by achieving CR, PR, or stable disease [4•]. Subsequently, the use of PCI has been considered for patients with ED-SCLC who respond to initial therapy. In 2014, the results of a Japanese clinical study were presented, which confirmed the usefulness of PCI for patients with ED-SCLC. This study showed that PCI reduces the incidence of brain metastasis in patients with ED-SCLC who achieve stable disease or a better response with initial therapy, but it does not prolong survival [5••]. In the 2014 edition of the *Guidelines for the Treatment of Lung Cancer* from JLCS, the use of PCI for patients with ED-SCLC has been changed from “recommended” to “not recommended,” and the guidelines only recommend PCI for patients with LD-SCLC who achieve CR after initial treatment (grade A recommendation) and patients with ED-SCLC who achieve CR after initial treatment (Grade B).

## PCI for Patients with CR

Auperin et al. performed a meta-analysis of individual data for a total of 987 patients who achieved CR after chemotherapy in seven randomized controlled studies [6–11] conducted between 1977 and 1995. PCI significantly reduced the cumulative incidence of brain metastasis, with a hazard ratio of 0.46 (95 % confidence interval [CI] = 0.38–0.57; *p* < 0.001), and the cumulative incidence of brain metastasis at 3 years after randomization was 58.6 % in the non-PCI group vs. 33.3 % in the PCI group. In addition, overall survival (OS) was significantly better in the PCI group compared with the non-PCI group (hazard ratio = 0.84; 95 % CI = 0.73–0.97; *p* = 0.01), with the 3-year-survival rate being 15.3 % in the non-PCI group vs. 20.7 % in the PCI group. Subgroup analysis revealed that the survival was longer in the PCI group regardless of age, performance status, initial disease stage, or type of induction therapy [3]. The longer survival (hazard ratio = 0.85; 95 % CI = 0.73–0.99) in patients with LD-SCLC (847 patients; 86 % of all subjects) seems to be a reliable finding, but the improvement of survival (hazard ratio = 0.77; 95 % CI = 0.54–1.11) in patients with ED-SCLC (140 patients; 14 %) requires further confirmation. In addition, the definition of CR differed among the trials, with some basing it on a plain chest x-ray film, while others required computed tomography (CT) of the chest or brain. The definition also differed from current clinical practice in Japan, where magnetic resonance imaging (MRI) of the head and sometimes positron-emission tomography (PET) are performed to confirm CR. Based on the above-mentioned reports, PCI is also considered for patients achieving good PR, in addition to those with CR.

## PCI for Patients with ED-SCLC

Slotman et al. conducted a prospective controlled study, in which 283 patients with ED-SCLC who showed some response to initial treatment were randomized to PCI group or non-PCI group. The primary endpoint was time to symptomatic brain metastases. The cumulative incidence of symptomatic brain metastases was significantly lower in the PCI group (hazard ratio = 0.27; 95 % CI = 0.16–0.44; *p* < 0.001), with the cumulative incidence at 1 year being 14.6 % in the PCI group and 40.4 % in the non-PCI group. Disease-free survival was also significantly longer in the PCI group than in the non-PCI group, being a median of 14.7 weeks vs. 12.0 weeks (hazard ratio, 0.76; 95 % CI, 0.59-0.96; *p* = 0.02). The proportion of patients with first recurrence in the brain was 9.1 % in the PCI group and 35 % in the non-PCI group. OS was significantly longer in the PCI group than in the non-PCI group, with the median survival time being 6.7 months vs. 5.4 months (hazard ratio, 0.68; 95 % CI, 0.52-0.88; *p* = 0.003). The 1-year survival rate was also higher in the PCI group than in the non-PCI group (27.1 % vs. 13.3 %) [4•].

However, in the protocol of this study, CT or MRI imaging of the brain should only be performed if the patient had symptoms suggestive of brain metastasis. Accordingly, the absence of brain metastasis was not confirmed by MRI (or CT) before chemotherapy or PCI, and brain metastases were not investigated after protocol treatment unless a suggestive symptom developed. There were several other concerns with this study, including variation of the radiation dose and fractionation schedule (20 Gy in five or eight fractions, 24 Gy in 12 fractions, 25 Gy in 10 fractions, and 30 Gy in 10 or 12 fractions) and induction chemotherapy with anticancer drugs other than platinum agents being given to some patients.

## PCI for Japanese Patients with ED-SCLC

In 2014, the results of a randomized, controlled phase III study investigating the efficacy of PCI in Japanese patients with ED-SCLC were reported to the American Society of Clinical Oncology (ASCO) and the European Society for Medical Oncology (ESMO). Patients with ED-SCLC who responded to platinum doublet chemotherapy were randomized to either PCI group (25 Gy in 10 fractions) or non-PCI group. The absence of brain metastasis was confirmed by MRI prior to enrollment. The primary endpoint was OS, while the secondary endpoints were the time to brain metastasis (checked every 3 months by MRI), progression-free survival (PFS), and safety. A sample size of 330 was planned, but the interim analysis found no superiority of the PCI group over the non-PCI group, so this study was terminated. The interim analysis was performed in 163 patients (84 in the PCI group and 79 in the non-PCI group), among whom 111 died. Many of the patients received initial chemotherapy with cisplatin + irinotecan, carboplatin + etoposide, or cisplatin + etoposide. Patients who achieved CR accounted for 12 % of the PCI group and 15 % of the non-PCI group. All patients in the PCI group received 25 Gy of radiation in 10 fractions, with a median treatment time of 14 days (range, 10 to 23 days). The incidence of brain metastasis was significantly lower in the PCI group (*p* < 0.001), with the 12-month cumulative incidence being 32.4 % in the PCI group and 58.0 % in the non-PCI group. New brain metastases were treated by radiation therapy in 31.3 % of the PCI group and 80.4 % of the non-PCI group. Among these patients, 3.2 % of the PCI group and 68.6 % of the non-PCI group underwent whole-brain irradiation, while 28.1 % of the PCI group and 19.6 % of the non-PCI group received stereotactic irradiation. The median PFS was comparable between the PCI group (2.2 months) and the non-PCI group (2.4 months), with a hazard ratio of 1.12 (95 % CI = 0.82–1.54). After disease progression was confirmed, second-line therapy was performed in 82 % of the PCI group and 89 % of the non-PCI group, while third-line therapy was performed in 43 % of PCI patients vs. 53 % of non-PCI patients and fourth-line therapy was performed in 16 and 27 %, respectively. Thus, a slightly higher percentage of patients in the non-PCI group received second-line or subsequent therapy. OS was longer in the non-PCI group than in the PCI group, being a median of 15.1 months vs. 10.1 months (hazard ratio = 1.38; 95 % CI = 0.95–2.02; *p* = 0.091). While the frequency of grade 2 or more severe adverse events was not increased in the PCI group, anorexia and discomfort were slightly more frequent in this group [5••].

In response to these findings, the 2014 edition of the *Guidelines for the Treatment of Lung Cancer* from JLCS changed the recommendation about PCI for patients with ED-SCLC from “recommended (Grade B)” to “not recommended (Grade D)”.

OS was shorter in the PCI group probably for the following two reasons, based on the data on posttreatment. (a) Only limited therapeutic options are available if brain metastases develop after PCI. In the non-PCI group, 68.6 % of the patients who developed brain metastases received whole-brain irradiation and could subsequently choose to receive stereotactic radiotherapy as is available after PCI. In Japan, patients are routinely monitored to detect brain metastasis, so performing PCI for prophylaxis may have limited value. (b) After PCI, patients tend to miss out on second-line or subsequent chemotherapy. Since PFS was comparable in the PCI group and the non-PCI group, administration of further chemotherapy after a relapse has marked influence on OS. After PCI, many patients complained of anorexia or taste disorders. This suggests that a decrease of performance status after PCI that does not show up in figures may have resulted in a lower percentage of patients receiving further chemotherapy, while it is unlikely that the non-PCI patients would miss the opportunity to receive chemotherapy under the Japanese health care system.

## Treatment Schedule of PCI (Doses and Fractionations)

According to a meta-analysis performed by Suwinski et al., PCI reduced the incidence of brain metastasis in a dose-dependent manner up to a total dose of 30 to 35 Gy [12]. Kotalik et al. also performed a meta-analysis and reported that delivery of 30–36 Gy in fractions of 2–3 Gy/day reduced the incidence of brain metastasis more effectively than 25 Gy in 10 fractions [13]. However, Le Pechoux et al. performed a randomized study in 720 patients comparing PCI at different doses and reported no significant difference in the incidence of brain metastasis between 25 and 36 Gy. They examined the subjects for brain metastasis by CT or MRI before enrollment, and patients were assigned to receive 25 Gy in 10 daily fractions or 36 Gy in either 18 daily fractions or 24 fractions with two sessions daily. There was no significant difference in the incidence of brain metastasis between the 25 Gy group and the 36 Gy group (hazard ratio = 0.80; 95 % CI = 0.57–1.11; *p* = 0.18), with the 2-year cumulative incidence of brain metastasis being 29 vs. 23 %. However, OS was significantly higher in the 25 Gy group (hazard ratio = 1.20; 95 % CI = 1.0–1.44; *p* = 0.05), with the 2-year survival rate being 42 vs. 37 %. Adverse events were slightly more frequent in the 36 Gy group, although there were no significant differences (malaise occurred in 30 % of the 25 Gy group and 34 % of the 36 Gy group, while headache affected 24 and 28 % and nausea/vomiting affected 23 and 28 %, respectively). Le Pechoux et al. concluded that PCI at 25 Gy should remain the standard treatment for LD [14]. They also assessed late neurotoxicity at 3 years after PCI in this study and reported that patients in both groups showed communication deficits, weakness of the legs, intellectual deficits, and memory deficits, with no significant difference of late neurotoxicity between the two groups [15]. Wolfson et al. also assessed the late neurotoxicity of PCI in patients with LD who achieved CR following initial treatment, comparing three doses and fractionation schedules (25 Gy in 10 daily fractions, 36 Gy in 18 daily fractions, and 36 Gy in 24 fractions twice daily). At 12 months after PCI, the incidence of neurological deterioration was 62 % in the 25 Gy/10 fractions group, 85 % in the 36 Gy/18 fractions group, and 89 % in the 36 Gy/24 fractions group, being significantly higher in the patients receiving 36 Gy. At 12 months, the incidence of chronic neurotoxicity was 60 % in the 25 Gy/10 fractions group, 85 % in the 36 Gy/18 fractions group, and 89 % in the 36 Gy/24 fractions group, also being significantly higher in the patients receiving 36 Gy (*p* = 0.02). Based on these findings, they concluded that 25 Gy is superior to 36 Gy from the perspective of late toxicity [16]. Based on the above findings, the *Guidelines for the Treatment of Lung Cancer* from JLCS recommends delivery of PCI at 25 Gy in 10 fractions to 36 Gy in 18 to 24 fractions (Grade B recommendation). The schedules frequently used in Japan are 25 Gy in 10 fractions or 30 Gy in 15 fractions.

With regard to the timing of PCI, Auperin et al. analyzed data on subgroups of patients in the above-mentioned meta-analysis and found a significant trend for a lower incidence of brain metastasis among patients who received PCI earlier after induction therapy. They also reported that survival was not prolonged when PCI was performed at 60 days or more after induction therapy. Accordingly, they proposed that PCI should be performed early after completion of induction therapy (within 6 months of its initiation) [3]. However, it has been reported that neurotoxicity is increased when PCI is performed in combination with chemotherapy [17], which suggests that PCI should at least not be delivered concurrently with chemotherapy.

## Toxicity of PCI

A Japanese phase III study of patients with ED revealed that alopecia, skin disorders, headache, anorexia, nausea, vomiting, dizziness, malaise, amnesia, etc. were reported as acute reactions to PCI [5••]. These reactions are usually treated symptomatically by anti-edema therapy with corticosteroids or Glyceol. Late effects of radiation, such as cognitive deterioration, are likely to occur at 6 to 24 months after PCI [18]. It was reported that toxicity becomes more severe as the individual fraction dose or total dose increases [19]. However, Grosshans et al. investigated the late effects at 2 years after PCI (25 Gy in 10 fractions) and reported no significant differences in any of the neurocognitive parameters after PCI compared with before PCI [20]. Thus, there is no consensus about the late effects of PCI. This may be partly because precise evaluation of the late effects of PCI is difficult due to the influence of tumor progression or chemotherapy.

## TRT for Patients with ED-SCLC

Slotman et al. performed a phase III study of TRT in patients with ED. Patients with ED-SCLC who responded to chemotherapy were enrolled. They were randomly assigned (1:1) to receive either TRT (30 Gy in 10 fractions) or no TRT. All underwent PCI. The primary endpoint was OS at 1 year in the intention-to-treat population. OS at 1 year was not significantly different between groups: 33 % (95 % CI = 27–39) for the TRT group versus 28 % (95 % CI = 22–34) for the control group (hazard ratio [HR] = 0.84, 95 % CI = 0.69–1.01; *p* = 0.066). However, in a secondary analysis, 2-year OS was 13 % (95 % CI = 9–19) versus 3 % (95 % CI = 2–8; *p* = 0.004). PFS was longer in the TRT group than in the control group (HR = 0.73, 95 % CI = 0.61–0.87; *p* = 0.001). The most common grade 3 or higher toxic effects were fatigue (4.5 vs 3.6 %) and dyspnea (1.2 vs 1.6 %). They concluded that TRT in addition to PCI should be considered for all patients with ED-SCLC who respond to initial therapy [21••]. It seems important to identify subgroups of patients who could benefit from this protocol in Japan and investigate whether performing PCI and TRT is useful for ED-SCLC also in Japan.

## Conclusion

**Fig. 1:**
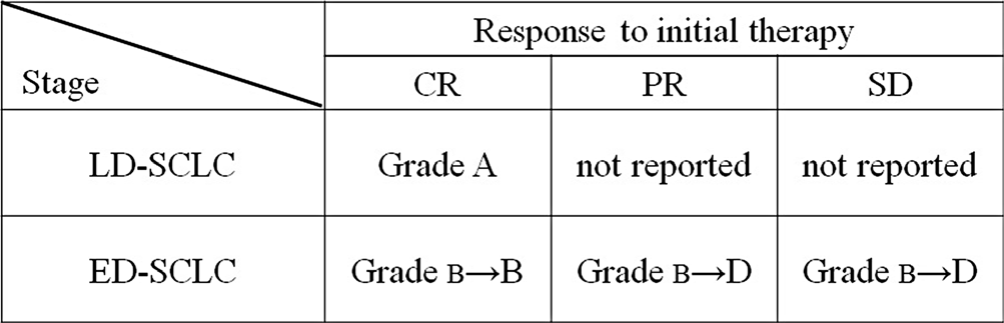
Indication for PCI in the 2014 edition of *The Guideline for the Treatment of Lung Cancer* from the Japan Lung Cancer Society. Grading of recommendations, *A* strongly recommended with high quality evidence. *B* Recommended with moderate quality evidence. *C1* Weakly recommended with low quality evidence. *C2* Negative recommended with low quality evidence. *D* Strongly negative recommended with high quality evidence.

After the phase III study conducted by Slotman et al., PCI was extended to patients with ED-SCLC. However, a Japanese phase III study failed to confirm the benefit of PCI for patients with ED-SCLC. All studies have demonstrated the effectiveness of PCI for preventing brain metastasis, this is not in doubt, but PCI seems to have a limited influence on OS. The current recommendations regarding PCI are summarized in Fig. 1 according to disease stage and the response to initial treatment. These recommendations raise many issues for the future, including the following 3 points.

- Is PCI necessary for patients with LD-SCLC who achieve CR or good PR after initial therapy? Since the effectiveness of PCI was not confirmed in patients with ED-SCLC, further investigation is needed to determine whether PCI is required for patients with LD-SCLC who achieve CR or good PR after initial therapy, although this is the current standard treatment. It would be interesting to determine whether PCI prolongs OS, as was found in the meta-analysis performed by Auperin et al. if patients are regularly screened for brain metastases (every 3 months during the above-mentioned Japanese phase III study in patients with ED-SCLC) after undergoing PET at the time of diagnosis and after initial treatment followed by PCI or observation alone.
- Is PCI necessary for patients with ED-SCLC who achieve CR after initial therapy? Since the phase III study conducted in Japan included patients with CR (12–15 %), the difference of OS in this group is a matter of concern.
- Is stereotactic radiotherapy (SRT) efficient for SCLC? There have been two retrospective analyses of the efficacy of SRT for brain metastasis in patients with SCLC. One demonstrated that SRT provided effective local tumor control in the majority of patients [22], while the other study found efficacy for metastases <2 cm [23]. If chemotherapy fails to achieve sufficient control, repeated SRT is often required for the treatment of recurrent brain metastases after initial SRT. Therefore, it is important to discuss how SRT should be incorporated into the treatment of SCLC. When the efficacy of SRT has been demonstrated, the significance of PCI should also be reconsidered.

Although there are several problems associated with PCI, it should at least be considered for patients with LD-SCLC who achieve CR following initial therapy, after giving the patient a detailed explanation of the advantages and disadvantages. However, there is no evidence justifying the use of PCI in elderly patients, patients with cognitive deterioration, or patients whose general condition is poor, because these patient populations are usually excluded from previous clinical studies. Appropriate selection of patients for PCI is very important. There have been recent reports on risk factors for the recurrence of brain metastasis after PCI [24] and about biomarkers of radiation neuropathy [25]. It is hoped that the characteristics of patients for whom PCI should be considered or avoided will be better defined in the future.
